# Heuristic bias in stem cell biology

**DOI:** 10.1186/s13287-019-1355-1

**Published:** 2019-08-07

**Authors:** Peter Quesenberry, Theo Borgovan, Chibuikem Nwizu, Mark Dooner, Laura Goldberg

**Affiliations:** 1 Division of Hematology and Oncology, Rhode Island Hospital, The Warren Alpert Medical School of Brown University, Providence, RI USA; 2 Division of Hematology/Oncology, Rhode Island Hospital, Center for Stem Cell Biology Research, Coro West, Suite 5.01, 1 Hoppin St, Providence, RI 02903 USA; 3 Center for Computational Molecular Biology, Brown University, Box 1903, 164 Angell Street, Providence, RI 02912 USA

**Keywords:** Stem cell hierarchy, Stem cell continuum, Representative heuristics, Availability heuristics, Heuristic bias

## Abstract

When studying purified hematopoietic stem cells, the urge for mechanisms and reductionist approaches appears to be overwhelming. The prime focus of the field has recently been on the study of highly purified hematopoietic stem cells using various lineage and stem cell-specific markers, all of which adequately and conveniently fit the established hierarchical stem cell model. This methodology is tainted with bias and has led to incomplete conclusions. Much of our own work has shown that the purified hematopoietic stem cell, which has been so heavily studied, is not representative of the total population of hematopoietic stem cells and that rather than functioning within a hierarchical model of expansion the true hematopoietic stem cell is one that is actively cycling through various differentiation potentials within a dynamic continuum. Additional work with increased emphasis on studying whole populations and direct mechanistic studies to these populations is needed. Furthermore, the most productive studies may well be mechanistic at the cellular or tissue levels. Lastly, the application of robust machine learning algorithms may provide insight into the dynamic variability and flux of stem cell fate and differentiation potential.

## Background/main text

Recently, in preparing an NIH grant application, in which we had some very intriguing novel data on how cell systems modified themselves, a colleague groaned, “This isn’t going to fly”. Innocently I rejoined, “Why not?”. His answer, “You know they’re going to demand mechanisms.” “This is the start of something potentially important. Besides mechanisms can come later and by the way mechanisms usually do not fully explain the workings of complex systems”, was my retort.

When studying purified cells, the urge for mechanisms and reductionist approaches appears to be overwhelming. This raises several questions: (1) How often is a determination of molecular mechanisms truly important? (2) How do you define mechanisms? (3) Can important fundamental or clinical research occur without the knowledge of mechanisms? (4) At what level are mechanisms operative; tissue, cellular, molecular etc.?

We have been focused on the hematopoietic stem cell field and our research has largely related to cellular mechanisms. This is a field in which the development of marrow stem cell transplantation has saved many thousands of lives. Interestingly, the initial supposed mechanism for action, the simple killing of leukemic cells with high-dose chemotherapy, was, at least partially, in error. A major part of the allogeneic transplant effect is an immune attack against the leukemia, not direct drug killing of the cells [1]. This field has been based on our knowledge of marrow stem cells and a huge effort has been ongoing to support mechanistic studies of these cells. The key initial step was purification of the stem cells from other cells in the marrow. This has largely been accomplished, or (at least) that is the common accepted dogma [2]. Billions have been invested in studying these purified cells, as to the mechanisms underlying their cellular life, death, and regulation. The results from this field have led to demands for purification in many other biological entities. Now in molecular studies, as to the specific action of molecules, some purification strategies are necessary. However, when applied to complex biologic systems or subsystems, the utility of purification needs to be questioned. We have a prime example with hematopoietic stem cells. The prime focus of the field has recently been on the study of highly purified hematopoietic stem cells, including lineage-negative c-Kit+Sca-1+CD150+ stem cells, and the elucidation of the molecular mechanisms underlying their fate. The cell is purified through a deletion of lineage marker-positive marrow cells followed by the selection for “stem cell epitopes,” i.e., c-Kit, Sca-1, and CD150 [2]. This results in a highly purified dormant population of stem cells which can renew hematopoiesis in lethally irradiated mice [1–4]. These cells may repopulate, and while they are non-cycling, dormant cells, even at this level of purification, the repopulations seen are quite variable; the cells are heterogeneous. Studying purified lineage-negative, rhodamine-low, Hoechst-low stem cells traversing cell cycle, synchronized under cytokine stimulation, we have also shown virtually total heterogeneity at different points in cell cycle [3]. This all leads up to the real shocker: the purified hematopoietic stem cell, which has been so heavily studied, is not representative of the total population of hematopoietic stem cells, at least in murine studies [4, 5]. The population of stem cells which exist in whole marrow is actively cycling, always changing, and cannot be “purified” by current standard epitope selection approaches. The problem here is that the purification discards the great bulk of cycling stem cells resulting in the isolation of a relatively rare non-cycling cell. A significant number of repopulating stem cells are present in the lineage-positive populations, which are often discarded with the standard purifications. Thus, the purified entity is not fully representative. The major focus of investigators has been on stem cell purification, since clearly this would allow for a definition of the true stem cell population, but this focus has been at the expense of consideration of other possibilities. This would appear to represent a classic example of representative and availability heuristics as described by Kahneman and Tversky [6]. Much data supported the purification approach: the obviousness of the hierarchical stem cell model with differentiation to end cells, thus, diminishing pursuit of alternative possibilities.

While the population as an entity can be defined, the individual entities cannot be defined. The individual variables may give you little, but the combination may be critically informative. This was elegantly pointed out by Till, McCulloch, and Siminovitich [7] in their description of colony-forming units among spleen colonies (CFU-s), an early stem cell candidate. They showed that the population was evaluable, but the individual stem cells were totally stochastic and heterogeneous. They compared this to radioisotopes in which the decay rates of individual isotopes were totally heterogeneous, but the half-lives of the population as a whole were very exact and reproducible. In biologic systems, purifications will almost always lead to potentially erroneous conclusions. Consider studying an individual wolf and then attempting to describe the behavior of the wolf pack. Similar analogies can be drawn for the study of individual bees or ants in trying to understand population behavior.

The reasons that investigators proceeded solely to “purify” stem cells and then study them without pursuing other possibilities lies in the apparent obviousness of the system and the elegant manner in which this model explained hematopoiesis. The following appeared to indicate that a hierarchical system with differentiation from primitive stem cells to terminally differentiated end cells constituted the hematopoietic system: the work with CFU-s, the observations of recovery of hematopoiesis after irradiation or cytotoxic marrow injury, the nature of erythroid repopulations in plethoric mice administered erythropoietic-containing agents, and the sequential repopulation of marrow and blood cells with marrow transplantation approaches. The observation that purified “dormant” hematopoietic stem cells would repopulate marrow and blood seemed to seal the argument. Thus, it was apparent that removing cells with surface epitopes restricted to differentiated cells would remove “contaminating” differentiated cells from marrow allowing a further purification of cells with defined stem cell antigens (c-Kit, Sca-1, and CD150). This became the dominating dogma in the field, and other possibilities were not explored. This constitutes a combination of the representative and availability heuristics as described by Kahneman and Tversky [6].

The population, or pack, would appear to be the critical biologic entity to study as opposed to the purified entity. The study of individual isolated cells may, of course, be informative, especially for specific cellular characteristics under specific experimental conditions; but the study of individual isolated cells will not wholly elucidate the nature of the cellular systems under consideration, nor will study of these cells in isolation provide comprehensive insight into biochemical or molecular mechanisms underlying the regulation of specific complex cellular systems. Recent advances in computational algorithms make it now possible to process large heterogenous datasets and gain meaningful biological insights. These would allow researchers to take a wholistic, systems-based look into stem cell biology. It also presents an opportunity to employ nearly unbiased investigations into the mechanistic studies of stem cell populations, and to capture constantly changing biological systems. A re-purposing of these technologies may help us quantitatively classify the extensive variation of a stem cell phenotype over time, and it may also provide insight into the dynamic variability and flux of stem cell fate and differentiation potential.

Thus, it would seem appropriate to reconsider the demands for cellular purifications in the study of cellular systems and to rethink the approach to the study of mechanisms underlying basic cellular phenomena. Reductionism is rarely helpful here.

## Conclusions

Mechanisms probably have to be considered on the complex mixed whole population of marrow (or other) cells. Eliminating the myriad of signals in the complex cellular mix probably limits most studies on purified stem cells with regard to deriving comprehensive regulatory insights. In addition, most mechanistic studies are directed at biochemical and molecular details, while the truly important mechanisms may be at the cellular or tissue levels.

A modest suggestion is to increase emphasis on studying whole populations and direct mechanistic studies to these populations. Furthermore, the most productive studies may well be mechanistic at the cellular or tissue levels.

## Data Availability

Not applicable.
